#  “COUNT YOUR AGE BY FRIENDS, NOT BY YEARS”: AN INTERPERSONAL PERSPECTIVE ON SUBJECTIVE VIEWS OF AGING

**DOI:** 10.1093/geroni/igac059.897

**Published:** 2022-12-20

**Authors:** Amit Shrira, Yoav Bergman, Anna Kornadt

## Abstract

Humans are social beings, and as we age, meaningful and close relationships become increasingly important. However, the literature on older adults’ subjective views of aging (VoA) tends to focus on the link between such views and health outcomes, overlooking important interpersonal correlates. This symposium consists of four presentations that focus on various concepts, study designs, and populations, and offers novel insights regarding how VoA operate in the social domain. The first presentation (Bergman et al.) establishes, using a diary study, the mediating role of understanding others’ emotional states (i.e., Theory of Mind) in the connection between VoA and positive social relationships. The second presentation (Mejía & Hooker) examines, through daily assessment, how VoA differentiate the processes by which older adults construct interactions with their closest social partners to progress toward health goals in daily life. The third presentation (Neupert & Can) employs a diary study and demonstrates that daily fluctuations in both subjective age and ageist attitudes are important determinants for older adults’ well-being. The fourth presentation (Shrira), a clinical study, focuses on how orthopedic patients’ rehabilitation is affected by their own and their healthcare professionals’ VoA. Together, the findings highlight the relevance of VoA to interpersonal functioning and health, and offer valuable information for both researchers and clinicians regarding the importance of this direction in future explorations of older adults’ VoA and physical/psychological well-being.

